# Supplementary material for: Eukaryote Genes Are More Likely than Prokaryote Genes to Be Composites
Source: Genes (Basel). 2019 Aug 28;10(9):648. doi: 10.3390/genes10090648 (PMC6769587; doi:10.3390/genes10090648)
Supplement: Supplementary file 1 [file genes-10-00648-s001.pdf]

Supplementary Materials

S1. Odds ratio (OR) test on GO annotations

To analysis the distribution of composite gene in eukaryotes and prokaryotes, apart from the COG annotations, we carried out the Odds ratio (OR) test on GO annotations as well. The results were collected from EggNOG output. Analysis is similar to COG analysis, for both eukaryotes and prokaryotes genes, counted the frequency of each function, OR value, upper and lower confidential interval (CI) values with conservative Bonferroni correction. Composite genes acted as cellular components and involved in biological process are statistical more likely to be from eukaryotes but genes in molecular function did not show much difference between eukaryotes and prokaryotes. The detailed information is showing in S1.

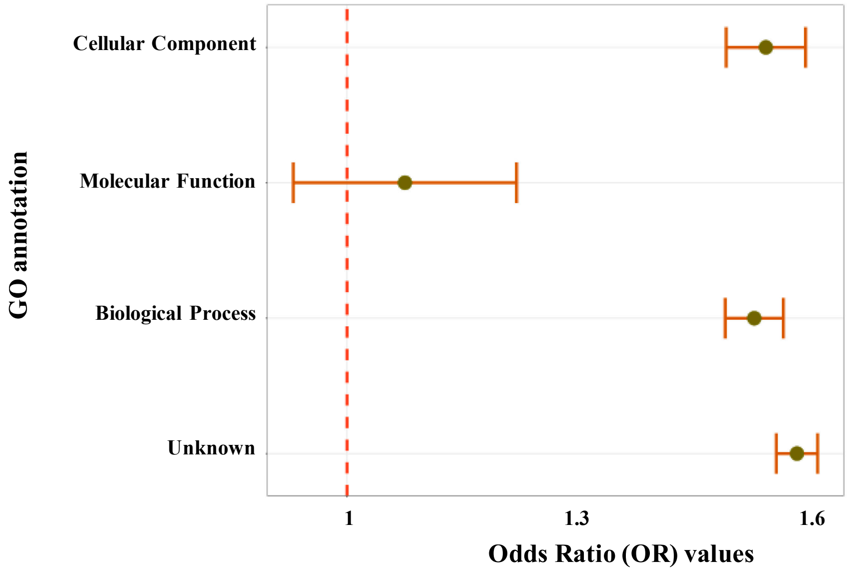

Figure S1. Numbers of OR, corrected upper 95% CI and lower 95% CI value across all GO annotations.

Table S1. Numbers of composite and non-composite genes from eukaryotes and prokaryotes in different functional categories, OR, corrected upper and lower 95% CI values.

Functional annotations		Composite		Non-composite		OR	Corrected lower 95% CI	Corrected upper 95% CI
		Eukaryotes (a)	Prokaryotes (c)	Eukaryotes (b)	Prokaryotes (d)			
COG	A	4633	27	11303	40	0.607	0.281	1.314
	W	1019	19	2174	44	1.085	0.461	2.556
	C	3408	5603	9049	19626	1.319	1.220	1.426
	L	4043	5159	8477	14734	1.362	1.261	1.472
	T	16220	3376	41878	13821	1.586	1.485	1.694
	J	4594	4213	12711	18724	1.606	1.490	1.731
	P	4674	4867	9861	16715	1.628	1.511	1.754
	E	4163	7247	7456	22038	1.698	1.578	1.826
	D	2620	638	6151	2620	1.749	1.499	2.041
	G	5291	4078	11275	15524	1.786	1.658	1.925
	Q	2905	1291	6917	5547	1.805	1.605	2.029
	N	89	535	304	3474	1.901	1.275	2.833
	B	2303	44	4606	169	1.920	1.132	3.259
	H	1954	3231	3633	11762	1.958	1.761	2.177
	I	4701	1919	10648	8966	2.063	1.876	2.268
	U	6011	814	16986	4758	2.069	1.824	2.346
	O	11432	2225	29759	12086	2.087	1.928	2.258
	F	1538	1777	3174	7748	2.113	1.863	2.396
	M	1916	3310	4139	15532	2.172	1.958	2.410
	V	826	1161	1906	6145	2.294	1.950	2.698
	K	8418	3372	22301	22575	2.527	2.358	2.709
	Z	4391	11	10486	91	3.464	1.290	9.304
	S	37613	9440	122130	125751	4.103	3.951	4.260
GO	Cellular Component	21063	9743	57403	41686	1.570	1.504	1.639
	Molecular Function	1832	1863	4469	4837	1.064	0.944	1.200

Biological Process	60444	16205	139926	58163	1.550	1.503	1.600
Unknown	58928	35835	283635	280001	1.623	1.588	1.660
